# ﻿ *Serralanguria*, a new genus of Languriini (Coleoptera, Erotylidae) from the Oriental Region, including one new species and one species transferred from the genus *Languria*

**DOI:** 10.3897/zookeys.1253.157027

**Published:** 2025-09-23

**Authors:** Zheng-Zhong Huang, Xing-Ke Yang, Si-Qin Ge

**Affiliations:** 1 Institute of Zoology, Chinese Academy of Sciences, Beijing, China Institute of Zoology, Chinese Academy of Sciences Beijing China; 2 University of Chinese Academy of Sciences, Beijing, China University of Chinese Academy of Sciences Beijing China

**Keywords:** Asian, Cucujoidea, key to genera, Languriinae, new combination, taxonomy

## Abstract

A new genus, *Serralanguria***gen. nov.** (type species: *Serralanguria sinensis***sp. nov.**), of the tribe Languriini from Asia is described and illustrated. This genus is characterized by a spherical, smooth pronotum, an elongate-oval terminal antennomere, and a terminal tarsomere distinctly broadened apically. On the basis of these morphological features, a new combination is proposed: *Serralanguria ochreipennis* (Fowler, 1890), **comb. nov.**, initially considered in *Languria* and later in *Caenolanguria* and *Anadastus*. A key to the genera of Languriinae known from China is provided.

## ﻿Introduction

Languriini Hope, 1840 is a tribe of the subfamily Languriinae, along with other two tribes Hapalipini Leschen, 2003 and Thallisellini Sen Gupta, 1968 (Leschen 2003). Among the three tribes, the species richness of Languriini is the highest, and is also the most problematic, for example, the distinctions between some genera are unclear, and the status of many monotypic genera needs to be re-examined. Since 2003, there has been limited taxonomic research on this tribe, including Robertson et al. (2004), Pal (2006), Huang et al. (2018), Huang et al. (2020), Toki (2020), and Mora-Aguilar and Delgado (2023). Currently, 13 genera and 89 species of the subfamily Languriinae have been recorded from China (Löbl and Smetana 2007; Huang et al. 2025). By examining the type materials of Languriinae deposited in the Natural History Museum, London, United Kingdom, and unnamed specimens in the collection of the Institute of Zoology, Chinese Academy of Sciences, a new genus and a new species were discovered and are formally described herein.

## ﻿Material and methods

The materials for this study are housed in the following collections:

**NHMUK**Natural History Museum, London, United Kingdom.

**IZAS**Institute of Zoology, Chinese Academy of Sciences, Beijing, China.

Specimens used in this study were relaxed in distilled water for 12–24 h prior to dissection of the genitalia. Detached parts were soaked in 10% KOH solution for 12–24 h at room temperature, then rinsed with distilled water, and dissected in 75% ethanol under a Nikon SMZ1000 microscope. All photographs were taken by a Canon EOS R5 digital camera equipped with a Canon MP-E 65 mm lens. The images were stacked using Helicon Focus v. 8.2.2 and edited in Adobe Photoshop CS6 to correct contrast, brightness and imperfections.

The holotype and paratypes of *S. sinensis* sp. nov. are deposited in the IZAS. The syntype of *S. ochreipennis* (Fowler, 1890), comb. nov. is deposited in the NHMUK.

Complete label data are listed for type specimens of the previously known species, using square brackets “[]” for our remarks and comments, “//” is used to separate data from different labels and a “/” is used to separate data from different lines of the same label.

Body length was measured from the apices of the mandibles to the apices of the elytra.

## ﻿Results

### ﻿Genus Serralanguria

Taxon classificationAnimaliaColeopteraErotylidae

Huang gen. nov.

29C9D26E-1373-5743-8E85-4E127BE8DD8A

https://zoobank.org/C363A821-C767-429F-A03C-7B51B95953A9

#### Type species.

*Serralanguria sinensis* sp. nov., by present designation.

#### Diagnosis.

This new genus belongs to the tribe Languriini based on the presence of a frontoclypeal suture and an antennal club composed of more than three antennomeres, and weakly asymmetrically dilated. *Serralanguria* gen. nov. can be easily distinguished from other langurine genera by its spherical and smooth pronotum, elongate-oval terminal antennomere, and terminal tarsomere distinctly broadened apically. The new genus is most similar to the genera *Anadastus* and *Caenolanguria* known from the Oriental Region, and in addition to the above-mentioned features, it can be distinguished from *Anadastus* by the rounded elytral apex and from *Caenolanguria* by the finely faceted eyes.

#### Description.

Length 5.0–7.0 mm. Body medium-sized, mostly glabrous, without metallic luster.

***Head*** subtriangular, frontoclypeal suture distinct, punctation fine and sparse; antennal insertions not visible from dorsal view; supraocular groove inconspicuous, bearing short yellowish hairs; vertex without stridulatory files. Antennae with 11 antennomeres, extending approximately to the elytral humeri, antennal club pubescence composed of six antennomeres, nearly circular in cross-section. Eyes small, prominent, finely faceted. Clypeus nearly rectangular; labrum densely covered with yellow setae anteriorly.

***Thorax*.** Pronotum spherical and convex, punctation fine and sparse, surface nearly smooth. Lateral and basal pronotal carina visible in dorsal view. Pronotum bending downward near the base, forming transverse fold. Basal foveae distinct but shallow. Lateral sides of pronotum curved; anterior angles rounded, posterior angles acute. Prosternal process elongate, apex straight. Procoxal cavities open. Scutellar shield heart-shaped, distinct; scutellary striole absent. Elytra with weakly impressed striate punctation becoming indistinct laterally and posteriorly; Elytral shoulders prominent; elytral epipleura distinct and gradually disappearing apically. Elytral apices rounded, without denticules. Legs slender; first tarsomere nearly as long as second; fourth tarsomere strongly reduced; terminal tarsomere dilated apically.

***Abdomen*** smooth, covered with dense yellowish setae. Ventrite I without divergent coxal lines.

#### Etymology.

The generic name is derived from two Latin roots: *serra* (“saw”) and *Languria* (generic name), in reference to the antennae of the type species, which bear a saw-like outline rather than being markedly enlarged.

#### Gender.

Feminine.

#### Distribution.

China, Malaysia, India, Philippines.

### ﻿ Serralanguria sinensis

Taxon classificationAnimaliaColeopteraErotylidae

Huang sp. nov.

E9AEF937-4195-5488-B6BA-C1AC9A91CDDB

https://zoobank.org/A26EA9CA-14A2-41AC-8771-6DB27D4F782E

Figs 1A–C, 2A–F

#### Type material.

**Holotype**: 1 ♂. China Guangxi Longsheng, Huaping Cujiang, 8 Aug. 2006, Meiying Lin leg.// Holotype [red label]; **Paratypes**: • 1 ♀. Same data as holotype; 1 ♀. Hainan Island Qiongzhong, 800 m, CAS//1980. Apr. 5// Paratype [yellow label].

**Figure 1.**
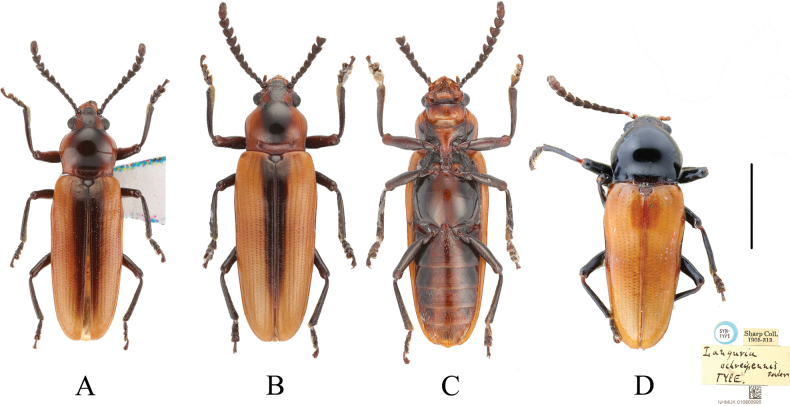
Type specimens of species of the genus *Serralanguria* gen. nov. A. *S. sinensis* sp. nov., male, holotype, dorsal view; B, C. Same, paratype, female; B. Dorsal view; C. Ventral view; D. *S. ochreipennis* (Fowler, 1890), comb. nov., syntype, dorsal view. Scale bar: 2 mm.

**Figure 2.**
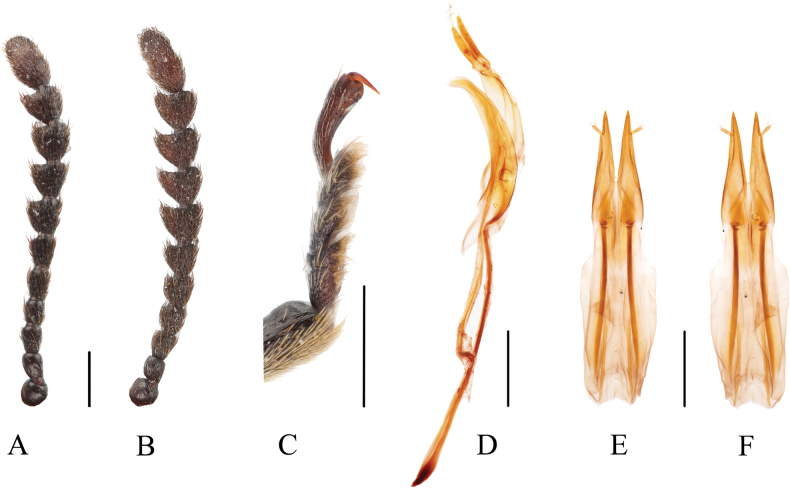
*Serralanguria sinensis* gen. et sp. nov. A. Holotype, male antenna; B. Paratype, female antenna; C. Holotype, male tarsus; D. Holotype, male genitalia; E. Paratype, female genitalia, ventral view; F. Same, dorsal view. Scale bars: 0.5 mm.

#### Other material.

**Figure 3.**
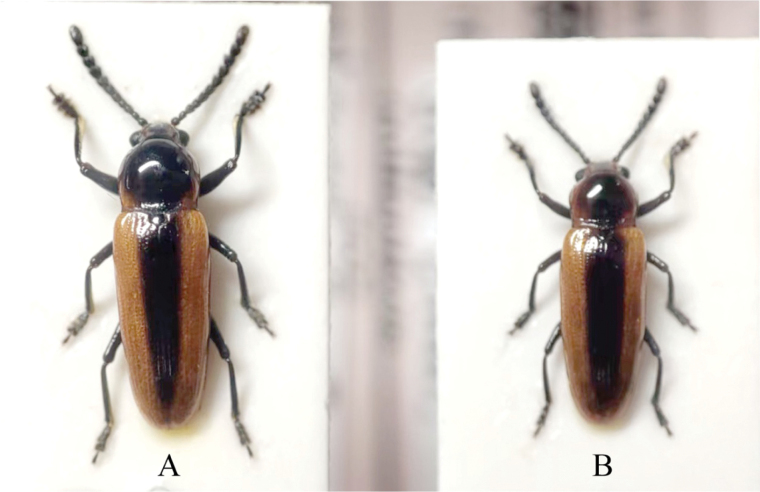
*Serralanguria sinensis* gen. et sp. nov., specimens from the private collection of Chen Zubin, collected in Pingyuan County, Guangdong Province, China. A. Female; B. Male.

1 ♂, 1 ♀, Guangdong Meizhou Pingyuan County, 15 Apr. 15 2024, alt. 1000 m, Zubin Chen, Liang Guo leg. (Private collection of Zubin Chen, Fujian, China, examined through pictures from the collectors) (Fig. 3A, B).

#### Diagnosis.

This species is readily distinguished from *Serralanguria ochreipennis* (Fowler, 1890), comb. nov. by its coloration: the pronotum is orange-brown with a continuous black median stripe extending from the anterior to posterior margin, whereas in *S. ochreipennis* comb. nov. the pronotum is subunicolorously black.

#### Description.

Body length 5.0–7.0 mm. Antennae and legs entirely dark brown or black. Body orange-brown, with one dark brown longitudinal stripe from middle of head, extending posteriorly along dorsal surface, but not reaching elytral apex, sometimes interrupted at pronotal base. Clypeus black. Ventral surface black with one longitudinal yellow stripe running from prosternum to abdominal apex. Dorsal surface glabrous; ventral surface bearing scattered short, decumbent setae.

***Head*** comparatively large; vertex nearly smooth, sparsely and weakly punctate, without setae, and short setae along anterior margin and around compound eyes; clypeus broadly protruding; gena prominently projecting anteriorly; gular suture deep and widely separated. Eyes prominent, finely faceted. Antennae elongate, exceeding elytral humeri; club six-segmented, segments mostly wider than long; terminal antennomere elongate-oval, covered with moderately dense setae.

***Thorax*.** Pronotum spherical and convex, slightly broader than long, nearly smooth and with fine, sparse punctation; lateral pronotal carinae visible dorsally; anterior angles rounded, posterior angles acute but not reaching elytral humeri. Basal foveae short, deep; basal margin complete, distinct. Prosternum finely punctate with yellow setae; prosternal process elongate, apex straight. Prohypomera bearing yellow setae near anterior angles. Scutellar shield narrowed at base and rounded at apex. Elytral surface with shallow, striate punctures arranged in regular rows; shoulders distinct, broader than pronotal base; apex tapering, rounded, not forming sutural angle. Mesoventrite finely punctate; metaventral median suture incomplete anteriorly, not reaching anterior margin of metaventrite. Abdomen finely punctate; ventrite 1 without postcoxal lines; last ventrite with dense yellow setae apically.

***Legs*** slender; terminal tarsomere thickened apically in both sexes.

***Genitalia*.** Aedeagus slender, elongate; median lobe gently curved in ventral view, apex abruptly deflexed; paramera slender, apically fringed with long yellow setae. Ovipositor short; coxites narrowly sclerotized, acute apically, bearing distinct styli with long setae.

#### Sexual dimorphism.

Pronotum in males more strongly convex and broadly rounded compared to females; terminal antennomere proportionally more elongate in females than in males.

#### Etymology.

The specific epithet, *sinensis*, is a Latinized adjective “Chinese.”

#### Gender.

Feminine.

#### Distribution.

China (Guangdong, Guangxi, Hainan).

#### Comments.

**Figure 4.**
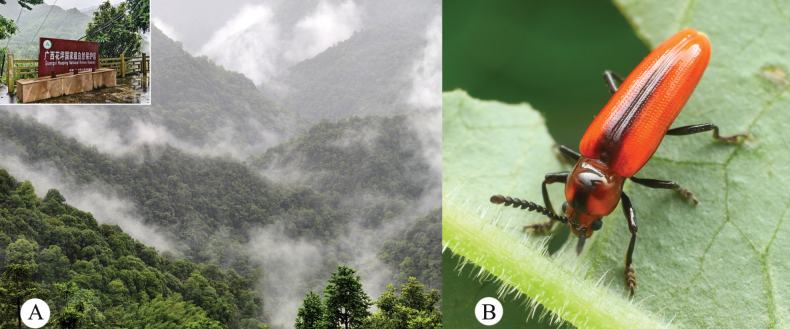
Habitat and habitus of *Serralanguria sinensis* gen. et sp. nov. A. Landscape of Huaping National Nature Reserve, Guangxi, China; B. Live habitus of *S. sinensis*, photographed in this place near the type locality.

Although the individuals shown in Figs 3A, B, 4B could not be examined directly, their morphological features, such as body shape, the distinctly elongate and oval terminal antennomere, and the characteristic coloration pattern, are fully consistent with those of the holotype and paratypes of *Serralanguria sinensis*. While some variation in overall coloration and the intensity of dark markings is observed among these individuals, no other known species of Languriinae exhibits the same combination of characters. Notable color variation between freshly collected and dried specimens is observable comparing the images in Figs 3, 4 and the specimens from the type series. A live individual was photographed in the field by Mr. Qian-Le Lu (Visiting Lecturer, Shenzhen University, China) on 20 June 2025 at Huaping Village, Guangxi, China, within the Huaping National Nature Reserve (Fig. 4A). The individual (Fig. 4B) displays a vivid orange-red coloration, whereas all type specimens preserved in the collection exhibit a duller orange-brown tone. The last-mentioned specimen was not collected but only documented by a photograph in the field. Therefore, the authors suppose that these three specimens with comparable body size to the type specimens can be interpreted as conspecific with the holotype of *Serralanguria sinensis* Huang, gen. et sp. nov.

### ﻿ Serralanguria ochreipennis

Taxon classificationAnimaliaColeopteraErotylidae

(Fowler, 1890) comb. nov.

C264D409-7B88-54AC-BB7C-C2DEC7F9EE06

Fig. 1D

Languria ochreipennis Fowler, 1890: 107. Type locality: Malacca. Type depository: NHMUK.
Coenolanguria ochreipennis : Fowler 1908: 17. (misspelling of Caenolanguria)
Anadastus ochreipennis : Arrow 1925: 233; Schenkling 1928: 24; Villiers 1945:132.

#### Type material examined.

**Syntypes**: 1 ♂, syntype [round label] // Sharp Coll. / 1905-313 // *Languria ochreipennis* Fowler TYPE. // QR Code NHMUK 010800995

#### Distribution.

India (Assam), Malaya Peninsula, Philippines.

#### Comments.

The syntype of *Anadastus ochreipennis* has all generic characters mentioned above, and therefore it is transferred to *Serralanguria*. They are as follows: pronotum globular and glossy, terminal antennomere elongated and oval-shaped, with tarsomere 5 markedly expanded at the apex.

### ﻿Key to genera of Chinese Languriinae

**Table:** 

1	Antennal club symmetric, cross-section round	**2**
–	Antennal club asymmetric, cross-section not round	**3**
2	Occiput without stridulatory file, antennae short, not exceeding base of pronotum, lateral sides of pronotum smooth (without denticles)	*** Microlanguria ***
–	Occiput with stridulatory file, antennae long, exceeding base of pronotum, lateral sides of pronotum with denticles (crenulate)	*** Neocladoxena ***
3	Elytral epipleuron​ distinct, reaching elytral apex	**4**
–	Elytral epipleuron absent or not reaching elytral apex	**9**
4	Distance between lateral margins of mandibles smaller than that between eyes	**5**
–	Distance between the lateral margins of the mandibles greater than that between eyes, particularly in females	*** Doubledaya ***
5	Eyes finely faceted	**6**
–	Eyes coarsely faceted	**8**
6	Subprognathous or hypognathous, clypeus not visible dorsally (or from above)	*** Neanadastus ***
–	Prognathous, clypeus visible dorsally (or from above).	**7**
7	Pronotum punctation extremely fine or absent; last antennomere elongate-oval; tarsomere apically thickened	***Serralanguria* gen. nov.**
–	Pronotum variably punctate; last antennomere shortly oval; tarsomere 5 slender, not thickened	*** Anadastus ***
8	Antennae short, generally not extending behind the base of pronotum.	*** Caenolanguria ***
–	Antennae long, reaching or exceeding the base of pronotum	*** Epilanguria ***
9	Pronotum without basal margin	*** Pentelanguria ***
–	Pronotum with basal margin	**10**
10	Antennae short, antennal club broad and compact; elytral surface lacking rugae	**11**
–	Antennae long, antennal club narrow and loosely segmented; elytral surface bearing rugae.	*** Paederolanguria ***
11	Antennomere 8 distinctly narrower than antennomere 9; lacinial apex with three teeth	**12**
–	Antennomeres 8 and 9 similar in width; lacinial apex with two teeth.	*** Megalanguria ***
12	Body slender; prosternal process weakly emarginate	**13**
–	Body robust; prosternal process strongly emarginate	*** Pachylanguria ***
13	Elytral apex truncate, with minute serrations	*** Tetraphala ***
–	Elytral apex acute, with outer angle projecting beyond sutural angle	*** Labidolanguria ***

## ﻿Discussion

The Oriental Region is notably rich in species diversity. According to Leschen and Węgrzynowicz (1998) and Leschen (2003), there are 30 genera that occur in the Oriental Region, of which 12 are monotypic. Despite the taxonomic complexity within the tribe, the distinctive morphological characters of *Serralanguria* gen. nov. clearly justify its establishment. In terms of morphological characteristics, the genus is rather similar and seems to be closely related to *Anadastus* and *Caenolanguria* distributed in the Oriental Region, while its spherical prothorax is similar to that of the genus *Penolanguria* distributed in Africa. The latter genus has a spherical pronotum, often covering part or all of the head, and its dorsal punctation is significantly coarser. The new combination is proposed herein because one species initially described in the genus *Languria* is finally transferred to *Serralanguria* gen. nov.

## Supplementary Material

XML Treatment for Serralanguria

XML Treatment for Serralanguria sinensis

XML Treatment for Serralanguria ochreipennis

## ﻿Additional information

### Use of AI

No use of AI was reported.

### Author ORCIDs

Zheng-Zhong Huang https://orcid.org/0000-0002-0085-6218

Xing-Ke Yang https://orcid.org/0000-0002-0870-4657

Si-Qin Ge https://orcid.org/0000-0001-5924-3400

### Data availability

All of the data that support the findings of this study are available in the main text.
